# Recombinant Paracoccin Reproduces the Biological Properties of the Native Protein and Induces Protective Th1 Immunity against *Paracoccidioides brasiliensis* Infection

**DOI:** 10.1371/journal.pntd.0002788

**Published:** 2014-04-17

**Authors:** Ana Claudia Paiva Alegre, Aline Ferreira Oliveira, Fausto Bruno Dos Reis Almeida, Maria Cristina Roque-Barreira, Ebert Seixas Hanna

**Affiliations:** Departamento de Biologia Celular e Molecular e Bioagentes Patogênicos, Faculdade de Medicina de Ribeirão Preto, Universidade de São Paulo, Ribeirão Preto, São Paulo, Brazil; University of California San Diego School of Medicine, United States of America

## Abstract

**Background:**

Paracoccin is a dual-function protein of the yeast *Paracoccidioides brasiliensis* that has lectin properties and *N*-acetylglucosaminidase activities. Proteomic analysis of a paracoccin preparation from *P. brasiliensis* revealed that the sequence matched that of the hypothetical protein encoded by *PADG-3347* of isolate Pb-18, with a polypeptide sequence similar to the family 18 endochitinases. These endochitinases are multi-functional proteins, with distinct lectin and enzymatic domains.

**Methodology/principal findings:**

The multi-exon assembly and the largest exon of the predicted ORF (*PADG-3347*), was cloned and expressed in *Escherichia coli* cells, and the features of the recombinant proteins were compared to those of the native paracoccin. The multi-exon protein was also used for protection assays in a mouse model of paracoccidioidomycosis.

**Conclusions/Significance:**

Our results showed that the recombinant protein reproduced the biological properties described for the native protein—including binding to laminin in a manner that is dependent on carbohydrate recognition—showed *N*-acetylglucosaminidase activity, and stimulated murine peritoneal macrophages to produce high levels of TNF-α and nitric oxide. Considering the immunomodulatory potential of glycan-binding proteins, we also investigated whether prophylactic administration of recombinant paracoccin affected the course of experimental paracoccidioidomycosis in mice. In comparison to animals injected with vehicle (controls), mice treated with recombinant paracoccin displayed lower pulmonary fungal burdens and reduced pulmonary granulomas. These protective effects were associated with augmented pulmonary levels of IL-12 and IFN-γ. We also observed that injection of paracoccin three days before challenge was the most efficient administration protocol, as the induced Th1 immunity was balanced by high levels of pulmonary IL-10, which may prevent the tissue damage caused by exacerbated inflammation. The results indicated that paracoccin is the protein encoded by *PADG-3347*, and we propose that this gene and homologous proteins in other *P. brasiliensis* strains be called paracoccin. We also concluded that recombinant paracoccin confers resistance to murine *P. brasiliensis* infection by exerting immunomodulatory effects.

## Author Summary

Paracoccin is a constituent of *Paracoccidioides brasiliensis*, a human pathogen that causes paracoccidioidomycosis, the most prevalent systemic mycosis in Latin America. Paracoccin is a dual function protein with domains for lectin and *N*-acetylglucosaminidase activities. Proteomic analysis of paracoccin preparation revealed its correspondence to a hypothetical protein from the *P. brasiliensis* isolate *Pb*-18, annotated as PADG-3347, which has a polypeptide sequence similar to family 18 endochitinases. These endochitinases are multi-functional proteins with distinct lectin and enzymatic activity domains. The multi-exon assembly of the correspondent gene (PADG-3347) was cloned and expressed in *E. coli*, and the physical and biological features of the recombinant protein was compared to those of the native paracoccin. Moreover, recombinant PADG-3347 was evaluated for its immunomodulatory properties and its ability to confer protection against murine *P. brasiliensis* infection. The results presented herein showed that mice treated with recombinant paracoccin displayed lower pulmonary fungal burdens and reduced pulmonary granulomas. These protective effects were associated with augmented pulmonary levels of IL-12 and IFN-γ.

## Introduction

*Paracoccidioides brasiliensis* [1], [2] is a thermo-dimorphic fungus that grows as a mycelium at 25°C and as a yeast at 37°C. *P. brasiliensis* infection in humans is known as paracoccidioidomycosis (PCM), a systemic granulomatous disease with high prevalence in Latin America [3]. An estimated 10 million people are infected with *P. brasiliensis* most people do not show clinical evidence of PCM.

The infection is acquired by inhalation of airborne propagules [4]. Once in the lungs, the fungus is stimulated by body temperature, and then it activates a number of genes that transform the conidia into the pathogenic form [5], [6]. Such dimorphism is an important feature of several pathogenic fungi, including *P. brasiliensis* [7]. The cell wall is involved in the morphogenetic changes that occur during this transition, and it plays key roles in the pathobiology of PCM [8].

Cell-mediated immunity is the main mechanism of defense against PCM [9], whereas a high humoral response correlates with disease dissemination [10]. Therefore, resistance to PCM is linked to high IFN-γ production by T helper type 1 (Th1) lymphocytes, and PCM susceptibility is associated with the lack of a cellular response and B cell activation [11], [12]. Early secretion of IL-12, followed by sustained secretion of IFN-γ, are important for resistance against *P. brasiliensis* infection [13]. The most physiologically important IL-12 target cells are T lymphocytes, which proliferate and differentiate into cells that produce type-1 cytokines, particularly IFN-γ [14].

We previously demonstrated that extracts of *P. brasiliensis* yeast contain an *N*-acetylglucosamine-binding lectin called paracoccin [15]. This protein binds to laminin in a sugar recognition-dependent manner, induces macrophages to produce TNF-α and NO, and is related to fungal growth [16] and morphogenesis [17]. In addition, paracoccin exerts *N*-acetylglucosaminidase activity [18]. Proteomic studies have showed that a paracoccin preparation requires a multi-domain protein, annotated as PADG-3347 (http://www.broadinstitute.org/annotation/genome/paracoccidioides_brasiliensis/GeneDetails.html?sp=S7000001960922883) that shares sequence similarity with the dual function proteins of chitinase family 18, and has an additional domain that contains the *N*-acetylglucosamine-binding activity [19]. These features explain the influence of paracoccin on fungal cell growth and morphogenesis, and its ability to interact with the host extracellular matrix and immune cells [20]. In this study, we cloned and expressed the putative recombinant paracoccin to compare the features of the recombinant protein to those of native paracoccin and to investigate the effect of administering the recombinant protein on an experimental model of PCM.

## Materials and Methods

### Ethics statement

The study was approved by the Ethical Committee for Ethics in Animal Research (CETEA) of the School of Medicine at Ribeirão Preto of the University of São Paulo. All animal experiments were conducted in accordance with the Ethical Principles in Animal Research adopted by the Brazilian College of Animal Experimentation (COBEA) (Protocol 20/2013-1).

### *P. brasiliensis* strain and accession number

The *P. brasiliensis* isolate used in this study, Pb18, was kindly provided by Dr. Roberto Martinez (Faculty of Medicine in Ribeirão Preto, University of São Paulo). Yeast cells were cultivated on Fava-Netto semisolid medium, YPD agar, and BHI broth. Virulence was maintained by consecutive intravenous infections in mice. The yeast were recovered from mouse lung tissue and then cultured on Fava-Netto medium at 37°C for 7 days. The viability of the yeast cells was determined by fluorescein diacetate and ethidium bromide staining [21], and it was always greater than 90%.

The nucleotide sequence of *PADG-3347*, hereafter called paracoccin, was assigned in the Broad Institute website as “PADG_03347.1 conserved hypothetical protein (Transcript:PADG_03347T0)” (http://www.broadinstitute.org/annotation/genome/paracoccidioides_brasiliensis/TranscriptDetails.html?component=%24DirectLink&service=direct&session=T&sp=S7000001960922884&sq=transcriptId%2C%2CS7000001960922884&sqpage=release%2FTranscriptDetail).

### Cloning of paracoccin

**Figure 1:**
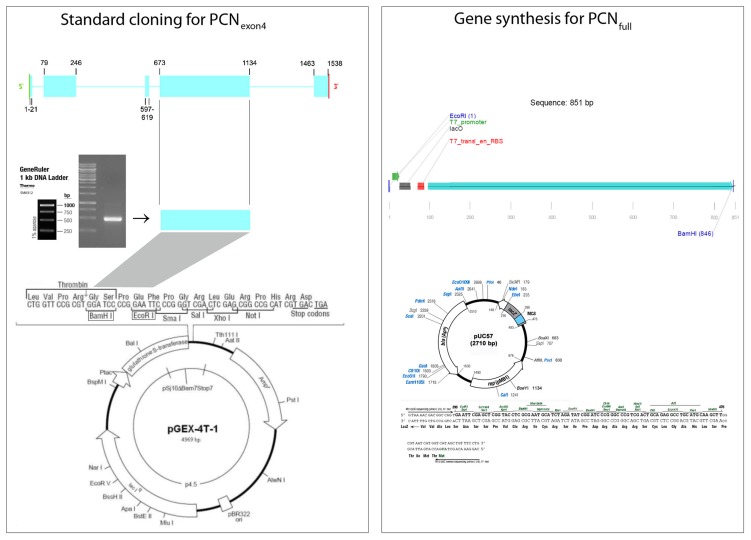
Cloning strategies for cloning the paracoccin ORF for expression. The left panel shows the standard strategy, with PCR amplification of the largest exon, restriction endonuclease digestion, and cloning into the expression vector pGEX-4T-1. The positions of the exons are displayed on the map of the gene. Genomic DNA template was extracted from *P. brasiliensis* strain Pb18. Agarose gel electrophoresis (mid-left) shows the corresponding band amplified by PCR. The exon 4 amplicon was cloned by *Bam*HI and *Eco*RI digestion. The right panel shows the strategy for synthesis of the predicted paracoccin sequence fused with the 5′-UTR elements for transcription in the vector pUC57. Green arrow, T7 promoter; black box, lacO (lac operator); and red box, the phage T7 trailer sequence for ribosome binding.

The gene encoding paracoccin is composed of five exons and four introns. Therefore, paracoccin was cloned in two different ways. First, the largest exon was amplified from *P. brasiliensis* genomic DNA using the oligonucleotide primers F_PADG_ (5′-CTGGATCCATGCAAGCACCCGACCAAC-3′) and R_PADG_ (5′-CGGGAATTCCTACCAACTCGTTATTGATAGAGCGATAA-3′), and then cloned into pGEX-4T-1 (GE Healthcare, Upsala, Sweden) flanked by *Bam*HI and *Eco*RI restriction sites. Second, the full-length, predicted ORF encoding paracoccin was synthesized, and cloned into the pUC57 vector (GenScript, Piscataway, NJ, USA). In the first strategy, the paracoccin fragment, referred to as rPCN_exon4_, is fused to a GST tag in the pGEX vector, whereas in the second strategy, the 5′-UTR region, which drives transcription of paracoccin (rPCN_full_) was also synthesized. Competent *E. coli* BL21 (DE3) cells were transformed with the recombinant vectors, and clones resistant to ampicillin were screened for the presence of the inserts by PCR. Positive clones were also submitted for DNA sequencing (ABI 3100, Applied Biosystems, Foster City, CA, USA). The two cloning strategies for the construction of the expression vectors are shown in figure 1. All clonings were performed using standardized methods [22].

### Protein expression and purification

#### rPCN_exon4_

*E. coli* BL21 (DE3) colonies transformed with pGEX-padg3347/exon4, were cultivated in 5.0 mL of LB medium with ampicillin (100 µg/mL). The culture was incubated overnight at 37°C with shaking (180 rpm). This pre-inoculum was diluted 1∶100 in 500 mL of the same medium, and incubated at 37°C with shaking (180 rpm) until the OD_600_ = 0.6. Then, IPTG was added to a final concentration of 0.1 mM. The culture was induced overnight at 21°C with shaking (80 rpm). Subsequently, the culture was centrifuged at 3,000× *g* for 20 min at 4°C, and then the bacterial pellet was resuspended in cold buffer A (Tris-HCl pH 7.5, 100 mM, 150 mM NaCl, 2 mM DTT, 10 mM EDTA, 0.2 mM sodium azide, and protease inhibitor), homogenized in Ovni Mixer 2000 at maximum speed for 4 pulses of 15 s at 60-second intervals on ice. The bacterial suspension was sonicated (5 pulses for 10 s each at 60-second intervals). Triton X-100 was added to a final concentration of 1%, and the lysed cells were centrifuged at 3,000× *g* for 30 min. The supernatant was collected and subjected to affinity chromatography on a glutathione-Sepharose 4B column equilibrated with buffer A and incubated with slow rotation for 1 h. Washings were performed with buffer B (buffer A plus 250 mM NaCl and 0.25% Tween-20), until the flow through had an OD_280_ of less than 0.002. The bound protein was eluted with buffer C (buffer A containing 10 mM reduced glutathione) and dialyzed against distilled water in an Amicon system (Amicon Division, W. R. Grace & Co., Beverly, MA, USA), using dialysis disks with nominal molecular weight limit (NMWL) of 10 kDa (Millipore Co., Billerica, MA, USA). The purified protein was quantified by using the method of Bradford [23].

#### rPCN_full_

*E. coli* BL21 (DE3) cells were transformed with pUC57-padg3347/fullORF, and plated on selective medium containing ampicillin. Recombinant strains were grown at 37°C with shaking until the OD_600_ reached 0.6. Recombinant protein expression was induced by the addition of IPTG at a final concentration of 0.5 mM. The cells were cultivated for an additional 5 h, and then harvested by centrifugation. The pellet was resuspended in lysis buffer (5 mM EDTA, 100 mM NaCl, 1 mM PMSF, and 50 mM Tris-HCl, pH 7.4) and the cells were disrupted by sonication. Triton X-100 was added to a final concentration of 1%, and the suspension was centrifuged at 3,000× *g* for 30 min. The homogenate, containing soluble and insoluble proteins, was diluted in 20 mL of urea buffer (8 M urea, 20 mM sodium phosphate, and 200 mM sodium chloride) and incubated overnight with agitation at room temperature. The homogenate was dialyzed against refolding buffer that contained 20 mM Tris, 100 mM NaCl, 0.2 mM PMSF, and various concentrations of urea to refold the protein. For refolding, the dialysis buffer was removed and replaced every 24 h, and with each replacement, the concentration of urea was reduced until the final dialysis contained no urea. After refolding, the protein was purified by affinity chromatography on an *N*-acetylglucosamine column, and the LPS was removed using an immobilized polymyxin B agarose column (BioRad, Hercules, CA, USA) according to the manufacturer's instructions.

### *N-*acetylglucosaminidase activity assay

The *N*-acetylglucosaminidase (NAGase) activity of the recombinant paracoccin was evaluated using 100 µL of p-nitro-phenol-*N*-acetyl-glucosaminidase (pNP-GlcNAc, 5 mM; Sigma), 350 µL of sodium acetate buffer (100 mM, pH 5.0), and 50 µL of each sample. To stop the reaction, 1 mL of 0.5 M sodium carbonate was added to the reaction tube, and then the absorbance was measured at 405 nm. The reaction conditions were the same as those used by Yabuki [24] and adapted by Ulhoa and Peberdy [25]. A unit of NAGase activity was defined as the amount of enzyme required to convert 1.0 mmol of *ρ*-nitrophenol per minute at 37°C. The specific activity was determined as the number of enzyme units per mg of total protein.

### Biotinylation of recombinant paracoccin

For biotinylation, 2.0 mg of recombinant paracoccin was incubated with 20 µL of a 1% aqueous solution of Ez-link Sulfo-NHS-LC-Biotin (Pierce Chemical Co., Rockford, IL, USA) for 2 h at 4°C in the dark. The biotinylated proteins were dialyzed against PBS, using a centrifugal filter device NMWL of 30 kDa (Millipore Co.), to completely remove the remaining biotin.

### Binding to laminin

Each well of a 96-well microplate (Maxi Sorp Fluoro Nunc, Roskilde, Denmark) was coated with 250 ng of laminin derived from mouse Engelbreth-Holm-Swarm sarcoma (EHS) (Sigma-Aldrich, St. Louis, MO, USA) diluted in carbonate buffer (pH 9.6) and incubated overnight at 4°C. Different concentrations of biotinylated protein were added to each well, and then the plate was incubated at room temperature (RT) for 120 min. The plate was then further incubated with neutravidin-peroxidase (Gibco BRL, Gaithersburg, MD, USA) for 90 min at RT. The binding of the biotinylated protein to laminin was detected by adding *O*-phenylenediamine dihydrochloride (OPD), and reaction was then stopped by the addition of 2 N sulfuric acid. Readings were made at λ = 490 nm (PowerWave X, Bio-Tek Instruments, Inc., Winooski, VT, USA). For the inhibition assays, biotinylated protein was pre-incubated with 100 mM *N*-acetylglucosamine, d-glucose, or d-galactose for 1 h at RT. Data shown are representative of three separate assays (reported in relative luminescence units, RLU).

### Production of TNF-α and NO

C57BL/6 mice were injected with 1.0 mL of sterile 3% sodium thioglycollate (Sigma-Aldrich). After 3 days, the animals were killed, and their cells were recovered by washing the peritoneal cavity with 5.0 mL of sterile PBS. The cells were immediately stored on ice and washed with RPMI 1640 medium (Flow Laboratories, Inc., McLean, VA, USA) containing 5% inactivated fetal calf serum (FCS) (Gibco BRL), and placed in 24-well culture plates (10^6^ cells/well). After incubation at 37°C 2 h, non-adherent cells were removed, and adherent cells were incubated in 5% RPMI medium containing rPCN_full_ or rPCN_exon4_ (250 ng/mL). TNFα and NO levels in the supernatants were measured by capture enzyme-linked immunosorbent assay (ELISA) and by nitrate accumulation, respectively, as previously described [15].

### Production of IgY antibodies

The procedure for immunizing chickens was performed according to Akita and Nakai [26], with slight modifications. Briefly, immunizations was performed by injecting (intramuscularly) 80 µg of native (PCN_prep_) or recombinant (rPCN_full_) paracoccin emulsified in Freund's complete adjuvant (Sigma-Aldrich). In weeks 2, 4, and 6 post-inoculation, booster immunizations were administered with antigens emulsified in Freund's incomplete adjuvant (Sigma-Aldrich). The eggs were collected and kept at 4°C. Eggs were also collected during the pre-immunization period to obtain antibodies used as control IgY (IgY irrelevant). The content of the yolk was diluted in ultra-pure water (1∶10), and after 6 h of gentle agitation at 4°C, high density lipids were precipitated by centrifugation (10,000× *g* for 25 min at 4°C) to collect the supernatant, which was rich in IgY. The IgY antibodies were purified by precipitation with 40% ammonium sulfate as previously described [27], [28]. The soluble fraction was collected and incubated at 4°C under constant agitation during the addition of ammonium sulfate. Then, the solution was centrifuged at 10,000× *g* for 10 min, and the first supernatant was discarded. The pellet was resuspended in PBS and 40% ammonium sulfate precipitation was repeated twice as described above. The last pellet was resuspended in PBS-azide and dialyzed against PBS using a membrane with NMWL of 10 kDa (Amicon Division, W.R. Grace Co.) to remove the ammonium sulfate.

### Confocal microscopy

The yeast cells were cultivated for 3 days at 37°C with shaking. The cells (10^6^ viable cells/mL) were fixed with 3.7% paraformaldehyde for 15 min, centrifuged at 3,000× *g* for 15 min, and resuspended again in 3.7% paraformaldehyde. After centrifugation, the cells were resuspended in PBS buffer with 0.05% Tween and 1% BSA (PBS-TB), containing the IgY polyclonal antibodies against anti-rPCN_full_ or anti-PCN_prep_ for 1 h at RT. Cells were then washed three times with PBS, and incubated for 1 h at RT with neutravidin AlexaFluor_488-conjugate (Life Technologies, Carlsbad, CA, USA) in PBS-TB for 1 h at RT in the dark. After 40 min, 10 µg/mL of calcofluor (Sigma-Aldrich) was added for the remaining 20 min of incubation. After washing with PBS, coverslips were placed onto the glass slides with Permount (Fisher Scientific, Pittsburgh, PA, USA) and fluorescence was analyzed using a fluorescence microscope. The slices were analyzed using a laser scanning confocal microscope (LSM-510 NLO; Carl Zeiss, Jena, Germany) using 40× NA 1.3 and 63× NA 1.4 plan apochromatic objectives. For phase-contrast microscopy studies, the samples were observed with an immunofluorescence microscope (DMI 4000B; Leica, Culver City, CA, USA). Z-series were processed with ImageJ (National Institutes of Health, Bethesda, MD, USA).

### Protection assay with recombinant paracoccin

#### Animals

The efficacy of protection with rPCN_full_ was assessed in four groups of five 6–8-week-old male BALB/c mice. They were bred and maintained under standard animal housing conditions in the animal house at the Ribeirão Preto School of Medicine, São Paulo University, Ribeirão Preto, SP, Brazil. All assays were performed in triplicate.

#### rPCN_full_ therapy and experimental infection

Each animal was injected subcutaneously (s.c.) with 0.5 µg of rPCN_full_ in 100 µL of sterile PBS. The treatment varied in terms of the number of rPCN_full_ injections (1–3), and the time intervals between injections (Table 1). The control animals were injected with vehicle only (PBS). The animals were then challenged by intravenous (i.v.) injection with 100 µL of a suspension containing 10^6^ *P. brasiliensis* yeast cells. The infection was evaluated on day 30 after challenge.

**Table 1:** Prophylaxis groups.

Groups	Treatment regimens (days before infection)
Control	PBS*
G1	17°, 10° and 3°
G2	10° and 3°
G3	10°
G4	3°

*Mice received PBS in a regimen similar to that of group 1.

#### CFU counting

Mice were killed by cervical dislocation on day 30 post-infection, and fungal burden in the lung was examined as previously described [29]. One lung per animal was aseptically removed, weighed, and the tissue was disrupted in 1.0 mL of sterile PBS using a tissue homogenizer (Ultra-Turrax T25 Basic; IKA Works, Inc., Wilmington, DE, USA). The number of viable cells was determined by plating crude and serially diluted homogenates onto brain heart infusion agar (BHI; HiMedia, Mumbai, Índia) supplemented with 4% (v/v) heat-inactivated fetal calf serum (FCS; Life Technologies, Camarillo, CA, USA) and with 96 µg/mL gentamicin (Gibco BRL). Plates were incubated at 35°C for 7–14 days. The number of *P. brasiliensis* colonies per gram of organ is reported as the mean and standard deviation (SD) of triplicate samples. Lung homogenate supernatants were also separated from the cell debris by centrifugation at 2,000× *g* for 15 min, and stored at −20°C for ELISA-based cytokine and nitric oxide (NO) quantification.

#### Histopathology

The lungs, obtained on day 30 post infection, were fixed in 10% formaldehyde for 24 h and embedded in paraffin. Tissue sections (5 µm) were stained with hematoxylin and eosin (H&E) and Gomori's methenamine silver stain and examined by light microscopy with an Axiophot photomicroscope (Carl Zeiss GmbH, Jena, Germany), coupled with a JVC TK-1270 camera (Victor Company of Japan Ltd., Tokyo, Japan).

#### Cytokine ELISAs

To determine the levels of IL-12p40, IL-12p70, TNF-α, IFN-γ, IL-4, and IL-10 in the supernatants of the lung homogenates, the levels of these cytokines were measured by capture ELISA with antibody pairs purchased from Pharmingen (San Diego, CA, USA). The cytokines were measured according to the manufacturer's protocol. The concentration of each cytokine was determined with reference to a standard curve generated by serial two-fold dilutions of recombinant murine cytokines.

#### NO production in the lungs

The concentration of NO_2_ was quantified by using the standard Griess reaction [30], by incubating 50 µL of supernatant from the lung homogenates with an equal volume of Griess reagent (1% sulfanilamide, 0.1% naphthylene diamine dihydrochloride, and 2.5% H_3_PO_4_), at RT for 10 min. NO_2_ was quantified by measuring the absorbance at 550 nm (Power Wave-X). Results are expressed as µM NO_2_ and the values were compared to a standard curve generated using known concentrations of NaNO_2._

### Statistical analysis of results

Statistical analyses of the differences between the means of experimental groups were performed using analysis of variance (ANOVA) followed by Tukey's test. Differences with *p* values less than 0.05 were considered statistically significant. All experiments were performed three different times, with a total of 5 animals in each group.

## Results

### Refolding and purification of the recombinant proteins

**Figure 2:**
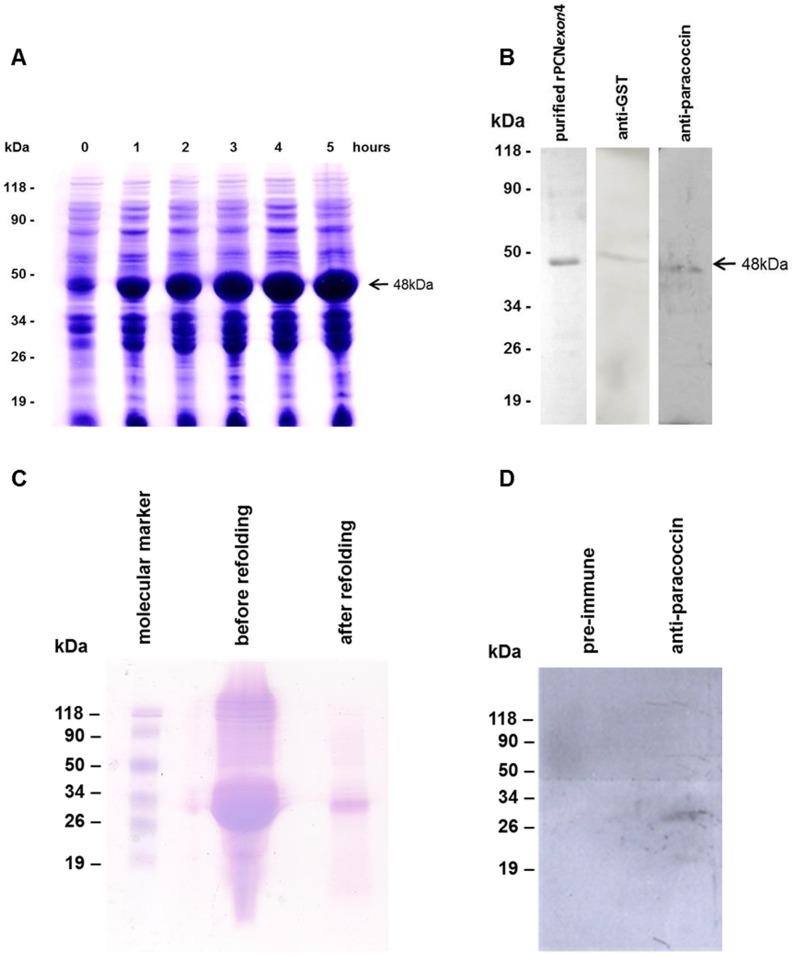
Electrophoretic profile of rPCN_exon4_ and rPCN_full_. **Panel A:** Induction time/response rPCN_exon4_ detected in the lysates of *E. coli.* The time elapsed since IPTG induction is shown in hours. **Panel B:** The bound material to glutathione-sepharose was separated by 12% SDS-PAGE Coomassie blue staining revealed a single band (lane 1), which was recognized by specific antibodies against GST (lane 2) and PCNprep (lane 3). **Panel C:** rPCN_full_ was purified and evaluated for its ability to bind to *N*-acetylglucosamine. The bound material was separated by 10% SDS-PAGE under reducing conditions, and then stained with Coomassie blue. Lane 1, material before refolding; Lane 2, material after refolding (a single band was detected with an apparent molecular mass of 28 kDa). Molecular markers were a mixture of pre-stained proteins (Fermentas). **Panel D:** The rPCN_full_ band was recognized by an anti-paracoccin antibodies (lane 2) and not by antibodies from pre-immune sera.

The predicted mRNA transcript sequence of the PADG_03347 gene was used to design the oligonucleotides for the cloning procedures. Recombinant *E. coli* strains were evaluated according to the expression of the largest exon of the paracoccin gene (rPCN_exon4_) and according to its predicted multiple exon assembly (rPCN_full_). After inducing the cells containing rPCN_exon4_ (Fig. 2A), the harvested bacterial cells were prepared and the resulting lysate was subjected to affinity chromatography on a column of glutathione-sepharose 4B. The bound protein was eluted with reduced glutathione, and was analyzed by SDS-PAGE, which showed a single, 48-kDa band. The predicted sizes of rPCN_exon4_ and the GST fusion tag are 22 kDa and 26 kDa, respectively. The band containing rPCN_exon4_ was recognized by anti-GST and anti-native paracoccin antibodies (Fig. 2B). In contrast, the induction procedure for rPCN_full_ generated highly enriched inclusion bodies, which were solubilized with urea and refolded. The refolded material was applied to a GlcNAc affinity column and, after elution with a specific sugar, the bound protein was analyzed by SDS-PAGE, which showed a single 28-kDa band (Fig. 2C) corresponding to rPCN_full_. The purified rPCN_full_ was also analyzed by western blotting. The 28-kDa band was recognized by anti-native paracoccin IgY (Fig. 2D). These results indicate that there is immunological identity between the native and recombinant forms of paracoccin, and that the GlcNAc-binding property was preserved after the refolding and purification processes.

### rPCN_exon4_ and rPCN_full_ reproduce the biological properties of native paracoccin

**Figure 3:**
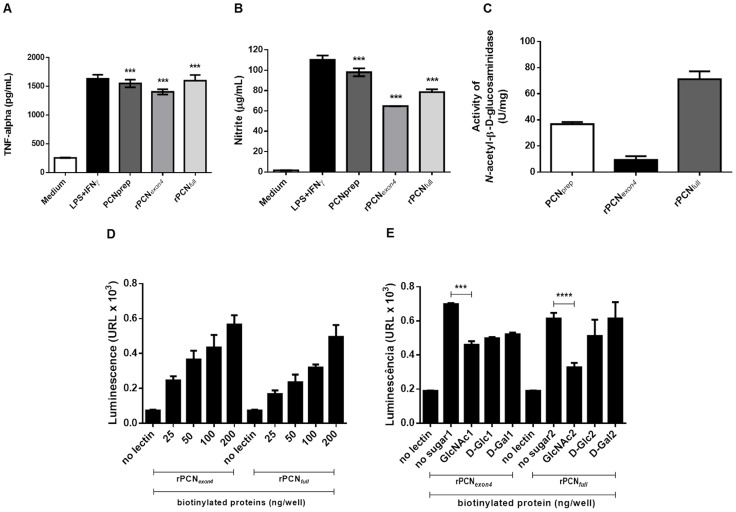
Biological and enzymatic properties of rPCN_exon4_ and rPCN_full_. **Panel A:** production of TNF-α, **Panel B:** production of NO by induced murine macrophages following *in vitro* stimulation with PCNprep and rPCN_exon4_ or PCNprep and rPCN_full_. Cells were harvested from the peritoneal cavity of C57BL/6 mice and induced with thioglycollate. Adherent cells were incubated for 48 h with different recombinant proteins (0.25 mg/mL), medium (negative control), or LPS+IFNγ (positive control). The standard deviation was calculated based on tests performed in triplicate. The activity of the samples was compared to that of the medium alone. **Panel C:** The PCNprep, rPCN_exon4_ and rPCN_full_ were assayed for NAGase activity. A colorimetric assay was performed in spectrophotometer set at λ = 405 nm. The standard deviation was calculated by analysis of experiments performed in triplicate. **Panel D:** Binding of rPCN_full_ to laminin. Different amounts of biotinylated recombinant protein were incubated with laminin (250 ng) coated in the microplate wells. The binding of the biotinylated protein was detected with a neutravidin-peroxidase conjugate and a chemiluminescent substrate. Luminescence readings are reported as relative luminescence units (RLU). **Panel E:** Inhibition of rPCN_full_ binding to laminin by sugars. Different concentrations of GlcNAc, d-glucose, and d-galactose were pre-incubated with the recombinant protein (100 ng), and the mixture was then added to the laminin-coated wells. The margin of error was calculated by analysis of triplicate experiments. Each sample with sugar was compared to a sample without sugar.

We previously reported that native paracoccin (PCN_prep_) induces the production of TNF-α and nitric oxide by murine macrophages and interacts with laminin in a sugar recognition-dependent manner [15]. PCN_prep_ also has *N*-acetyl-*β*-d-glucosaminidase activity, which probably accounts for the function of paracoccin during fungal growth and morphogenesis [18]. Comparison of the predicted polypeptide sequence of PADG_03347 with a database of functional domains using a Hidden Markov Model (HMM), HHPred, indicated that the enzymatic and lectin activities could be contained in its largest exon (exon 4). As shown in Fig. 3, rPCN_exon4_, rPCN_full_, and PCN_prep_ can induce murine peritoneal macrophages to produce TNF-α (Fig. 3, panel A) and nitric oxide (Fig. 3, panel B) at levels close to that of the positive control (LPS+IFN-γ). The NAGase activity of PCN_prep_, rPCN_exon4_, and rPCN_full_ was also evaluated (Fig. 3, panel C). The rPCN_full_ preparation showed an enzymatic activity of 0.054 U/mL and a specific activity of 2.65 U/mg. PCN_prep_ showed an 0.041 U/mL enzymatic activity and a specific activity of 2.05 U/mg. In contrast, the rPCN_exon4_ preparation had only residual NAGase activity, which was four times lower than that of PCN_prep_ (panel C).

We also assessed the ability of both rPCNs (at concentrations of 25–200 ng/well) to bind laminin. Our results show that rPCNs were able to bind laminin in a dose-dependent manner (Fig. 3, panel D). Moreover, this interaction was selectively inhibited by the presence of *N*-acetylglucosamine (Fig. 3, panel E), as was previously reported for PCN_prep_ [15].

These results have given us some clues about the structure and function of paracoccin, and showed that the lectin domain is preserved in both recombinant proteins. However, rPCN_exon4_ did not have enzymatic activity. This impairment of the enzymatic activity of rPCN_exon4_ led us to further characterize only rPCN_full_.

### Detection of paracoccin in yeast cells using IgY against the recombinant protein

**Figure 4:**
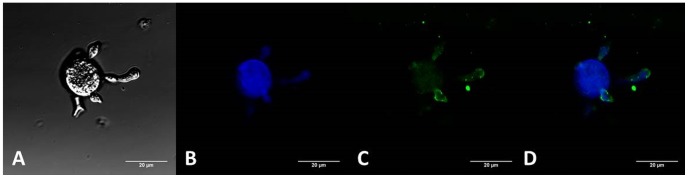
Anti-rPCN_full_ antibody reactivity on the yeast cell surface. Fluorescence labeling with anti-rPCN_full_ (**Panel A–D**) was evenly distributed over the yeast cell surface, with more intense labeling in some budding regions. Panel D is the merge of panels B and C.

To verify whether IgY anti-rPCN_full_ was able to recognize PCN_prep_ in its natural context, we performed an immunofluorescence assay with non-permeabilized *P. brasiliensis* yeast cells. As shown in figure 4, fluorescence labeling was mostly detected on the cell surface, particularly on daughter cells. A pseudohyphal form, seen in the preparation, was prominently labeled at its tip. This labeling was verified both in the yeast and hyphal forms of *P. brasiliensis* (data not shown), and reproduces previous results obtained with murine IgG raised against PCN_prep_.

### Effect of rPCN_full_ administration on pulmonary fungal load and histopathology

**Figure 5:**
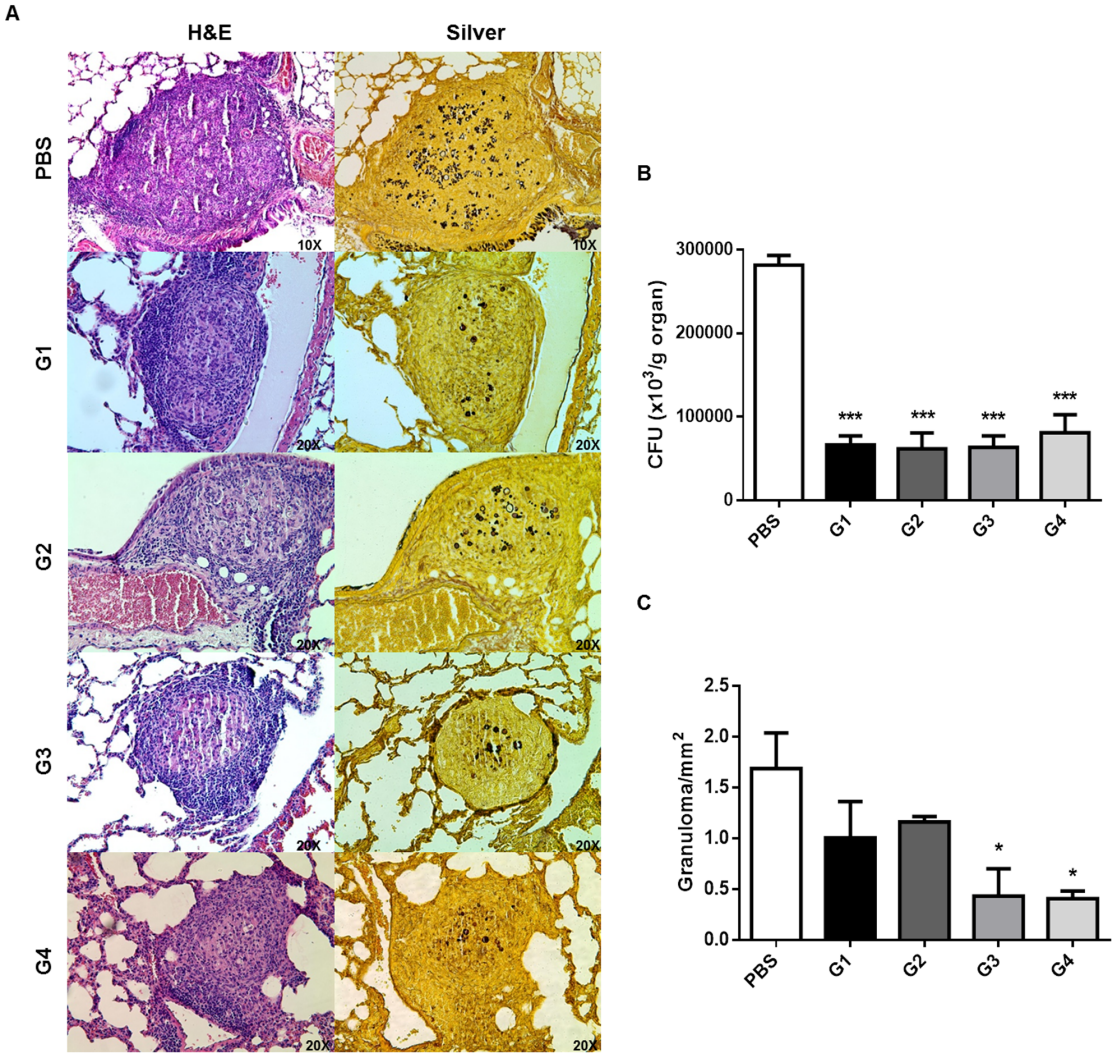
Fungal burden, granuloma incidence, and histopathology in infected mice that received prophylactic administration of rPCN_full_. Mice were infected (i.v.) with 10^6^ *P*. brasiliensis yeast cells and analysis was performed on day 30 after infection. **Panel A:** Pulmonary CFU recovery. Each group of five mice was either not treated (injected with vehicle [PBS]) or prophylactically treated according to protocol G1, G2, G3, or G4. **Panel B:** Morphometric analysis of lung sections in terms of granulomas/mm^2^ of tissue, and according area of granuloma. Bars depict the means and SD. *P<0.05 *versus* the PBS group. **Panel C:** Lung histopathology of infected mice that received prophylactic administration of rPCN_full_. Mice were infected (i.v.) with 10^6^ *P. brasiliensis* yeast cells and analysis was performed on day 30 after infection. The panels show representative lung sections from mice that were infected and not treated (PBS), or infected and prophylactically treated according to protocol 1 (G1), protocol 2 (G2), protocol 3 (G3), and protocol 4 (G4).

We evaluated whether prophylactic administration of rPCN_full_ affected the course of PCM using a murine model. To determine the most effective administration regimen, groups of BALB/c mice were administered a variable number of injections spaced at different time intervals (see Table 1, Materials and Methods), and then challenged i.v. with *P. brasiliensis* yeast. Thirty days after infection, pulmonary fungal burden lowered by approximately 80% in all rPCN_full_-treated groups compared to the untreated control group (Fig. 5B). Light microscopy examination of lung sections from all rPCN_full_-treated mice, regardless of the administration regimen, showed that the organ architecture was mostly preserved, with scant compact and well-organized granulomas and a few silver-stained yeasts (Fig. 5A). In contrast, coalescent granulomas were frequently observed in the lung sections of mice in the control group. Notably, a large amount of yeast were observed both inside and outside the granulomas. Morphometric analysis revealed significantly (*p*<0.05) lower granuloma density in the lungs of mice from group 3 (0.92 granulomas/mm^2^) and 4 (0.94 granulomas/mm^2^), which were injected with rPCN_full_ on day 10 and day 3 before infection, respectively, compared to the other treated groups (Fig. 5C). These results suggest that administration of rPCN_full_ could diminish the pulmonary damage in *P. brasiliensis* yeast-challenged mice.

### Pulmonary levels of cytokines and NO in challenged mice

**Figure 6:**
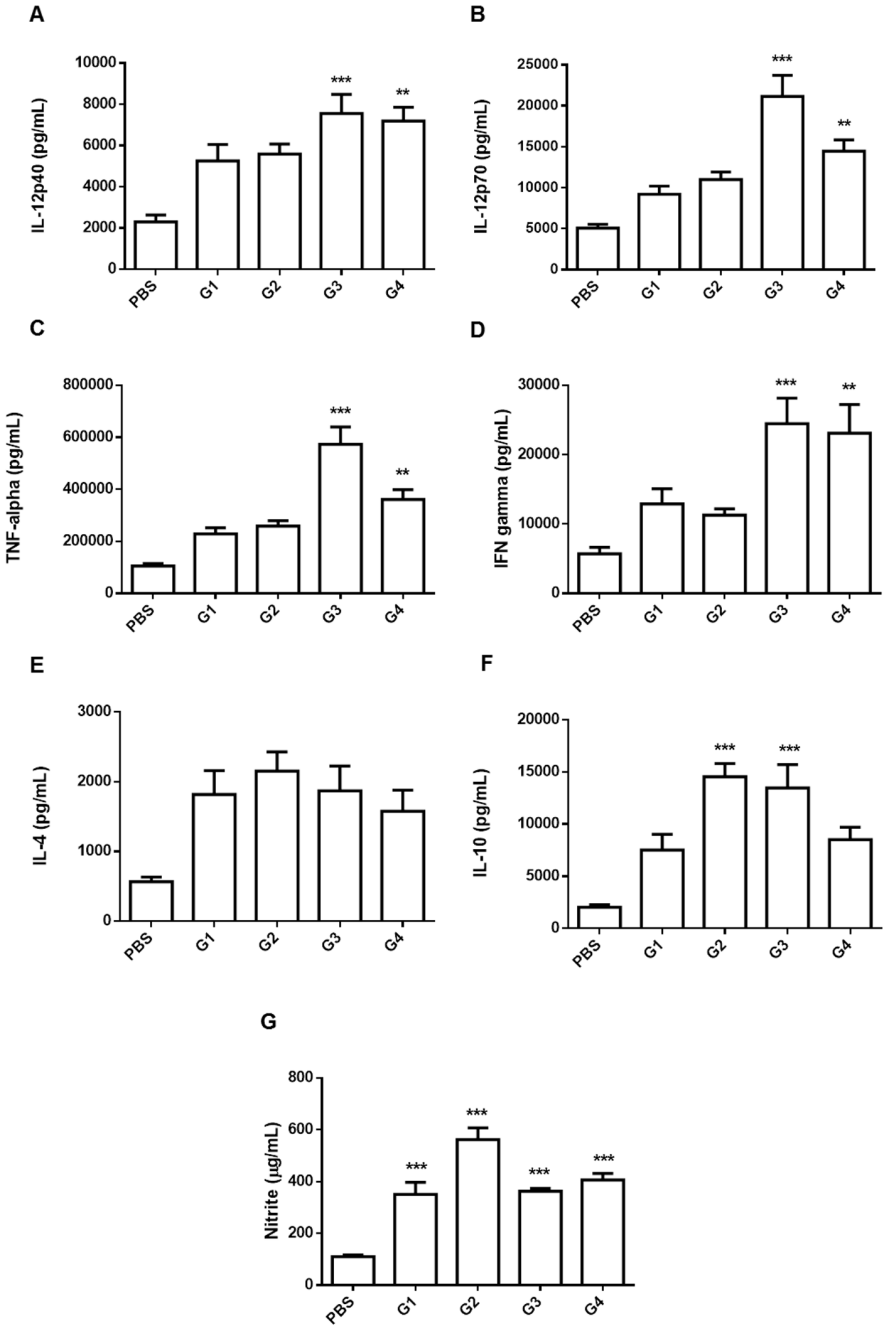
Prophylactic administration of rPCN_full_ increases proinflammatory cytokines and NO production. Mice were infected (i.v.) with 10^6^ *P. brasiliensis* yeast cells and analysis was performed 30 days after infection. IL-12p40 (A), IL-12p70 (B), TNF-α (C), IFN-γ (D), IL-4 (E), IL-10 (F), and NO (G) levels were measured in lung homogenates. Data are reported as the mean and SD of five mice per group and the experiments were performed in triplicate. *P<0.05 *versus* the PBS group.

To determine if the advantageous effect of rPCN_full_ administration was associated with induction of a protective immune response to fungal infection, the levels of cytokines and nitric oxide in lung homogenates were measured on day 30 after the challenge. The levels of IL-12p40 were three times higher in groups 3 and 4 than in the control group (Fig. 6A). Consistently, the levels of IL-12p70 were four and three times higher in groups 3 and 4, respectively (Fig. 6B). The production of TNF-α (Fig. 6C) and IFN-γ (Fig. 6D) was five and two times higher in mice of group 3, respectively, and approximately two and three times higher in group 4, respectively, than that of the control group. Therefore, mice that received rPCN_full_ following the protocols for groups 3 and 4, presented significantly higher levels of Th1 cytokines than the control mice. This inflammatory profile was strengthened by the detection of higher levels of pulmonary NO in animals that received rPCN_full_ as prophylactic therapy (Fig. 6G).

Regarding Th2 cytokines, IL-4 pulmonary levels in the treated and control groups of mice were not significantly different (Fig. 6E). In contrast, IL-10 levels were significantly higher in groups 2 and 3 (Fig. 6F). These results indicate that prophylactic administration of rPCN_full_ may confer protection against *P. brasiliensis* infection through induction of T-helper 1 immunity in the host. rPCN_full_ injection three days before the challenge appears to be the most favorable administration protocol, since the Th1 response might be balanced by high levels of pulmonary IL-10, which may prevent tissue damage caused by inflammation.

## Discussion

We demonstrated that the recombinant form of paracoccin (rPCN_full_) reproduced the biological activities of the native protein (PCN_prep_) and conferred remarkable protection against fungal infection, probably due to skewed Th1 immunomodulation.

Native paracoccin, referred to as PCN_prep_, was originally reported as a constituent of the GlcNAc-binding preparation (GNBP), an enriched extract of *P. brasiliensis* yeast, was shown to bind laminin [15], exert NAGase activity [18], and induce macrophages to produce TNF-alpha and nitric oxide [15], [18]. These activities were putatively attributed to a major 70-kDa protein in the GNBP fraction. This assumption is now tested frequently by newer proteomic and molecular studies. The lectin containing feature and enzymatic activities of PCN_prep_ could be attributed to the 28-kDa conserved hypothetical protein of *P. brasiliensis*, a double domain lectin/chitinase, whose gene is annotated in the Broad Institute website as PADG_03347.1.

Two products of the PADG_03347.1 gene, the proteins encoded by the largest exon (rPCN_exon4_) and by the multi-exon assembly (rPCN_full_), were both able to bind laminin and induce the production of inflammatory mediators by macrophages. However, the NAGase activity exerted by PCN_prep_ and rPCN_full_ was not reproduced by rPCN_exon4_. This enzymatic activity plays important roles in the biology of the fungus [17]. However, it remains to be determined if this enzymatic activity is also relevant to the fungal interaction with host immune cells. For this reason, rPCN_full_ was used in the subsequent *in vivo* experiments because it more accurately corresponded to the native protein.

Motivated by previous studies showing that paracoccin has interesting effects on immune cells [15], and that lectins are potential immunomodulatory agents [31], [32], we investigated the effect of recombinant paracoccin (rPCN_full_) administration on the course of murine PCM. The protocols were designed based on prophylactic administration of the plant lectin ArtinM in mice, which succeeded in conferring resistance to murine leishmaniasis [33] and PCM [34]. In our study, rPCN_full_ was administered to groups of mice at various schedules and time intervals. We found that prophylactic administration of rPCN_full_ conferred protection against PCM, and was associated with the development of Th1-immunity and a clear reduction of the disease severity. All the rPCN_full_ administration protocols led to significantly lower frequencies of pulmonary granulomas and decreased fungal burdens. The control mice, which received only vehicle, had high fungal loads and extensive granulomatous lesions that disseminated to the liver and spleen. The pattern of disease in mice treated with rPCN_full_ was very similar to that exhibited by mice treated with ArtinM [34] or P10 vaccine [35]. This pattern was also similar to the mild form of human PCM, which is easily cured by anti-fungal therapy [36], [37].

Mild forms of human PCM are consistently demonstrated to be associated with Th1-biased immunity, a response that is considered essential for conferring resistance in animal models of fungal diseases [13], [38]–[40]. In a different way, Th2 immunity is associated with severe and disseminated forms of fungal diseases. These features are well established not only for the experimental models of paracoccidioidomycosis [13], [41], but also for cryptococcosis [42], and candidiasis [38], [43]. By comparing the cytokine concentrations in lung homogenates from the control and rPCN_full_-treated mice, we could demonstrate that, among the groups that were treated with rPCN_full_, the mice that received a single injection on day 10 or day 3 before the challenge had the highest levels of Th1 cytokines and higher NO levels. Surprisingly, no exacerbated inflammatory lesions were observed in any of the rPCN_full_-treated animals, a fact that can be justified by the high levels of IL-10 in their lungs. The cytokine levels allow us to hypothesize that rPCN_full_ interacts with glycosylated receptors on the surface of immune cells and triggers the release of IL-12, as was demonstrated in mice treated with ArtinM, because it is well known that IL-12 stimulates NK and TCD4 cells to produce high levels of IFN-γ, which, in turn, induces high levels of NO and other proinflammatory mediators. They improve the fungicidal activity of macrophages [12], which is essential to confer resistance to *P. brasiliensis* infection. It was previously demonstrated that *in vivo* depletion of IFN-γ leads to exacerbation of lung infection and systemic dissemination of fungal cells [44]. In contrast, TNF-α increases the permeability of the vascular endothelium, macrophage accumulation in the infected tissues, the development of epithelioid granulomas and restriction of fungal spreading [45]–[47]. Granuloma formation as well as the local immune response played by phagocytic cells and sustained granulomatous inflammation depend on TNF-α synthesis and are necessary to restrict fungal growth and dissemination [48], [49]. Increased pulmonary levels of IL-10 were detected in animals that received rPCN_full_ on days 10 and 3 before challenge, and in animals that received rPCN_full_ only on day 10 before challenge. Although IL-10 is considered a major mediator that facilitates the infection [50], it is also able to prevent inflammatory injuries due to exacerbated Th1 immunity [51]. This mechanism also seems to be the case for the *P. brasiliensis*-infected mice, following inoculation with rPCN_full_, reported herein, or with ArtinM, as reported by Coltri [52].

Finally, our results support the relevance of the fungal protein paracoccin for the fate of experimental PCM. Prophylactic administration of a recombinant form of paracoccin resulted in a drastic reduction of pulmonary lesions and fungal burden compared to control mice. Our findings bring new insights into modulation of the immune response during *P. brasiliensis* infection with recognizable implications in the clinical investigation of PCM.
